# Local and Distant Dysregulation of Synchronization Around Interictal Spikes in BECTS

**DOI:** 10.3389/fnins.2017.00059

**Published:** 2017-02-10

**Authors:** Emilie Bourel-Ponchel, Mahdi Mahmoudzadeh, Patrick Berquin, Fabrice Wallois

**Affiliations:** ^1^Institut National de la Santé et de la Recherche Médicale U 1105, GRAMFC, CURS, CHU Amiens Picardie - Site SudSalouël, Amiens, France; ^2^Fonctional Exploration of the Pediatric Nervous System, CHU Amiens Picardie - Site SudSalouël, Amiens, France; ^3^Neuropediatry Unit, CHU Amiens Picardie - Site SudSalouël, Amiens, France

**Keywords:** BECTS, time-frequency analysis, interictal epileptic spikes, desynchronization, hypersynchronization, pre-spike

## Abstract

**Objective:** High Density electroencephalography (HD EEG) is the reference non-invasive technique to investigate the dynamics of neuronal networks in Benign Epilepsy with Centro-Temporal Spikes (BECTS). Analysis of local dynamic changes surrounding Interictal Epileptic Spikes (IES) might improve our knowledge of the mechanisms that propel neurons to the hypersynchronization of IES in BECTS. Transient distant changes in the dynamics of neurons populations may also interact with neuronal networks involved in various functions that are impaired in BECTS patients.

**Methods:** HD EEG (64 electrodes) of eight well-characterized BECTS patients (8 males; mean age: 7.2 years, range: 5–9 years) were analyzed. Unilateral IES were selected in 6 patients. They were bilateral and independent in 2 other patients. This resulted in a total of 10 groups of IES. Time-frequency analysis was performed on HD EEG epochs around the peak of the IES (±1000 ms), including phase-locked and non-phase-locked activities to the IES. The time frequency analyses were calculated for the frequencies between 4 and 200 Hz.

**Results:** Time-frequency analysis revealed two patterns of dysregulation of the synchronization between neuronal networks preceding and following hypersynchronization of interictal spikes (±400 ms) in the epileptogenic zone. Dysregulation consists of either desynchronization (*n* = 6) or oscillating synchronization (*n* = 4) (4–50 Hz) surrounding the IES. The 2 patients with bilateral IES exhibited only local desynchronization whatever the IES considered. Distant desynchronization in low frequencies within the same window occurs simultaneously in bilateral frontal, temporal and occipital areas (*n* = 7).

**Significance:** Using time-frequency analysis of HD EEG data in a well-defined population of BECTS, we demonstrated repeated complex changes in the dynamics of neuronal networks not only during, but also, before and after the IES. In the epileptogenic zone, our results found more complex reorganization of the local network than initially thought. In line with previous results obtained at a microscopic or macroscopic level, these changes suggested the variability strategies of neuronal assemblies to raise IES. Distant changes from the epileptogenic zone in desynchronization observed in the same time window suggested interactions between larger embedded networks and opened new avenues about their possible role in the underlying mechanism leading to cognitive deficits.

## Introduction

Benign Epilepsy with Centro-Temporal Spikes (BECTS) is the most common form of idiopathic childhood epilepsy with a prevalence of 15% in epileptic children aged 1–15 years (Panayiotopoulos et al., 2008). BECTS, more common in boys, with a sex ratio 6/4 (Wirrell, 1998) is characterized by brief, hemifacial sensori-motor seizures that typically originate in the centro-temporal area (Beaussart, 1972; Loiseau and Beaussart, 1973) associated with interictal epileptic spikes (IES) localized in the unilateral and/or bilateral centro-temporal areas, which are typically activated by drowsiness and slow non-Rapid eye movement (REM) sleep (Beaumanoir et al., 1974). The characteristics of the IES in BECTS constitute a clinical biomarker of this epilepsy. Well-defined IES in BECTS patients can be modeled by single tangential dipole sources oriented from central to frontal lobes and localized in the central regions (supra-sylvian) (Ishitobi et al., 2005). Despite the infrequent seizures and the focality of IES in BECTS, cognitive, and/or behavioral disorders have been repeatedly reported in BECTS patients (Vannest et al., 2015). An implication of IES in cognitive deficits has been suggested, but the underlying neurophysiological mechanisms remain poorly understood.

The mechanisms that propel neurons to the hypersynchronization of the IES are multiple, involving synaptic and non-synaptic interactions (De Curtis and Avanzini, 2001) and changes in the immediate cellular configuration (Manoochehri et al., 2017) and hemodynamic environment (Jacobs et al., 2009b; Osharina et al., 2010). Based on intra-cerebral multi-unit recording in refractory epilepsy, Keller and collaborators suggested that the interictal epileptiform activity in patients with epilepsy is not a simple paroxysm of hypersynchronous excitatory activity, but rather represents interplay of multiple distinct neuronal types within complex neuronal networks (Keller et al., 2010). IES would result from complex interactions within different neuronal populations whose activities decrease or increase not only during the IES but also before and after the IES by a few 100 ms (Keller et al., 2010). At a macroscopic level, EEG and fMRI studies have demonstrated that IES are associated with complex network interaction not only inside the epileptogenic zone but also in distant areas including the frontal and temporo-occipital cortices (Cataldi et al., 2013; Fahoum et al., 2013; Adebimpe et al., 2015a,b, 2016) with extensive functional (Besseling et al., 2013; Tang et al., 2014; Adebimpe et al., 2015a,b, 2016) and structural changes (Garcia-Ramos et al., 2015) in cerebral activities.

BECTS patients are not accessible to intra-cerebral multiunit recording. To address the complexity of neurophysiological mechanisms around the IES in BECTS, scalp HD EEG in the time-frequency domain was analyzed. We expected that, like in refractory epilepsies, (Keller et al., 2010; Jacobs et al., 2011), IES are associated with complex changes in synchronization, which could precede IES. Moreover, because time analysis of scalp HD EEG allowed analyzing cortical activity more globally, at a macroscopic level, we thought that TFR could contribute to a better understanding of the interaction between the epileptogenic zone and distant areas.

The present study was designed to investigate the dynamics of large-scale neuronal networks by non-invasive analysis of changes in synchronization around the IES based on time-frequency analysis of High-Density electroencephalography (HD EEG) in a homogeneous male population of BECTS children.

## Materials and methods

### Patients

Eight male patients with typical BECTS (mean age: 7.2 years, range: 5–9 years) were included in the study. BECTS was diagnosed on the basis of a typical clinical history and the presence of characteristic IES on standard EEG, according to ILAE criteria (Berg et al., 2010).

Clinical diagnostic criteria of BECTS included children presenting sensori-motor seizures with inconsistent secondary generalization, with an age of onset between 4 and 10 years (Beaumanoir et al., 1974) and typical diphasic spikes either isolated or occurring in clusters, unilaterally or bilaterally, in the centro-temporal areas on a standard normal background EEG (Beaumanoir et al., 1974). Patients with an abnormal neonatal history, intellectual deficit (IQ < 70), neurological abnormalities on physical examination, and/or any lesions in brain neuroimaging were not included in the study.

### Ethical considerations

The study was approved by the local ethic committee (CPP Nord-Ouest, No. A00782-39). Written Informed consent to participate in the study was obtained from the parents and all patients before inclusion.

### EEG acquisition

High-Density EEG were acquired with Ag/AgCl electrodes (*n* = 64), disposed according to the 10/10 international system (EasyCap®). The EEG was recorded by eemagine EEG software (eemagine Medical, Imaging Solutions GmbH, Berlin, Germany) and sampled at 1024 Hz (ANT Inc., Enschede, The Netherlands), in DC mode. Only a notch filter (50 Hz) was applied. A mastoid reference was used for acquisition.

HD EEG recordings were performed during quiet arousal. The electrode impedances were kept below 5 kΩ. The signals were re-referenced to an average reference for further analysis. Patients were monitored for movements during the acquisition, so that altered data could be later excluded.

### EEG analysis

#### Interictal epileptic spike selection and artifact rejection

For IES selection, artifact rejection and all subsequent analyses, data were arithmetically re-referenced to average reference. Because, the spike complex BECTS can be explained by one tangential source (Pataraia et al., 2008; Kakisaka et al., 2011), we used scalp potentials. Indeed, tangential sources are likely located in fissures and sulci, thus deeper than radial sources. Surface Laplacians are more sensitive to radial than tangential dipoles. Sources in sulci will be therefore minimized and further reducing their contribution to a surface Laplacian. Moreover, the scalp surface Laplacian, which may be interpreted as a method which minimizes volume-conduction effects (important for connectivity analyses), does require accurate spline interpolation and may be sensitive to the choice of the spline parameters (He, 1999).

Visual selection of IES containing similar spatial distribution, shape, and morphology is difficult and is associated with errors. To increase the accuracy of selection, IES were semi-automatically selected after reviewing the EEG with BESA® software.

First, typical BECTS spikes, characterized by diphasic or triphasic patterns distributed in the centro-temporal areas were selected manually (Beaumanoir et al., 1974). Second, IES sets containing similar spatial distribution, shape, and morphology were automatically selected by BESA® software. We used a 75% correlation cut-off between the search and target patterns with a reasonable range from 60 to 90%. To clearly identify focal spikes emerging from the noisy EEG background signals, a bandwidth of 2–70 Hz was applied.

EEG “IES epochs” were defined to the last 1 s before and after the IES peak (T0). Non-overlapping IES epochs lasting 2 s (comprising 2,048 data points) were considered for each spike set, to allow sufficient surrounding background (baseline) activity for analysis.

Single IES epochs were inspected for artifact contamination. Individual rejection criteria were based on the distribution of IES epochs in terms of mean amplitude and gradient (first temporal derivative) values. IES Epochs that were contaminated with artifacts after visual correction, representing a total of 15%, were rejected. Only IES epochs with background amplitude less than 200 μV were considered. Artifact-free IES epochs were then submitted to the source localization and time-frequency spectral analysis steps described below.

At the end of the marking process, the selected IES epochs were overlapped in a single diagram to confirm that they presented similar shapes. Finally, all steps of IES selection and artifact rejection were manually reviewed by two experienced neurophysiologists (FW, EB).

A mean of about 100 spikes per patient were finally analyzed.

#### Interictal spike source localization

To assess the homogeneity of the BECTS population, the sources of the average IES were estimated. To allow further analysis, the sources of IES must be located, for all patients, along the central sulcus with a tangential orientation and an anterior positivity of the dipole (Ishitobi et al., 2005). Due to the lack of a standardized validation method across source localization methods in clinical studies and considering the intrinsic limitation of each source modeling, different approaches may help clarify the nature of the presumed cortical source better than one approach can alone. Dipole and distributed EEG source localization are complementary and both methods are widely used to localize BECTS activity (Kamada et al., 1997; Lin et al., 2003; Huiskamp et al., 2004; Ishitobi et al., 2005; Pataraia et al., 2008; Kakisaka et al., 2011). Therefore, in our study, in addition to dipole based model (Dipole fitting), we used an algorithm named “Standardized Shrinking LORETA-FOCUSS” (SSLOFO) that integrates the two techniques (i.e., sLORETA and FOCUSS).

SSLOFO is an approach to combine the advantages of both low- and high-resolution methods in an automated fashion. Starting from a very smooth estimate, SSLOFO improves the spatial resolution using the recursive strategy of FOCUSS.

Artifact-free IES epochs were averaged and filtered with a 1 Hz high-pass filter (high pass: 6 dB/octave, zero phase) (Herrendorf et al., 2000; Ochi et al., 2000; Lantz et al., 2003; Groening et al., 2009; Elshoff et al., 2012). A standardized finite element head model (FEM) created from an averaged head of 50 individual MRIs in Talairach space (BESA Research® template), was used. This head model provided a realistic approximation of three compartments (brain, skull, and scalp) and was applied with the conductivity parameters of Scalp 0.33 S/m, Skull 0.0042 S/m, and Brain 0.33 S/m.

#### Time-frequency analysis

To characterize more precisely changes in neuronal activity occurring during each selected epoch, time-frequency analysis was performed, for frequencies between 4 and 200 Hz and including phase-locked and non-phase-locked representations to the IES.

To accomplish this goal, analysis were performed in 3 steps (pre-processing, time frequency representation, and statistical analysis) described in the following outlines.

##### Pre-processing

Non-overlapping IES epochs lasting 2000 ms were considered for each IES. A first relative baseline segment lasting 400 ms (−1000 to −600 ms before T0) was defined for each channel on each IES epoch. Time-frequency analysis was performed on the window between −1000 and + 1000 ms around T0.

To take into account a possible baseline selection effect, a second analysis was performed for 2 patients (patients 2 and 4) considering a larger and more distant reference period (−3000 to −1000 ms before T0). Time-frequency analysis was performed on the window between −3000 and +1000 ms around T0.

During the time-frequency averaging process, IES epochs were averaged without filtering to maintain the full bandwidth for time-frequency processing.

##### Time-frequency representation (TFR) (Figure 1)

**Figure 1:**
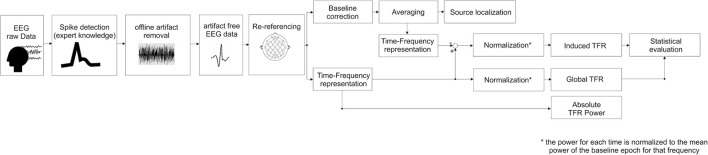
**Block diagram summarizing the steps followed in this study, (i.e., source localization and time-frequency representation processing, see text for details)**.

TFR was performed according to the procedures described by Hoechstetter et al. (2004) and implemented in BESA Research®. This procedure is able to distinguish global representations including phase-locked and non-phase-locked representations and extract induced representations corresponding to non-phase-locked activity (Figure 1).

#### Global time-frequency representation (G_TFR_):

TFRs were first computed on each selected IES epoch at each frequency. This method precisely identified the nesting principles that specifically underlie the IES activity. To establish the regional specificity of these findings, TFRs were extended to include all EEG channels.

TFRs were computed by applying complex demodulation (Papp and Ktonas, 1977). For each frequency of interest *f*_0_, the following three steps were performed:

1. The original time-domain signal (i.e., not subjected to any offline filtering) was multiplied by *sin(2πf*_0_*f)* and *cos(2πf*_0_*f)*, respectively. This modulation operation shifts every signal at frequency *f* to the difference and sum frequencies (*f* ± *f*_0_) in the frequency domain.
2. The resulting two signals were low-pass filtered to extract the frequency range originally centered around *f*_0_ and that was shifted to the low frequency range (*f* − *f*_0_). Thus, the low-pass cut-off frequency sets half of the width of the frequency band for which the envelope amplitude and phase is computed.
3. The two output signals of step (2) define the real and imaginary part of a complex signal as a function of time. Its magnitude corresponds to half of the envelope amplitude.

The time-frequency representation was calculated over each IES epoch. Frequencies were sampled (Gaussian filter) in 2 Hz steps and latencies were sampled in 25 ms steps, corresponding to a time-frequency resolution of ±2.83 Hz and ±39.4 ms at each time-frequency bin (full width at half maximum).

The TFRs of EEG activity was compared to the baseline segment, lasting 400 ms (−1000 to −600 ms before T0 of each IES) for all the patients and to the second baseline (−3000 ms to −1000 ms before T0 of each IES) for the patients 2 and 4. Because the amplitude of human surface EEG waves is in the range of 10 to 100 μV (Tong and Thakor, 2009), the averaged power of EEG baseline ongoing activity period [P_baseline_(f) below] over about 100 IES epochs never goes to zero or near zero. Also, TFRs was expressed as the relative power change to baseline activity at a time-frequency bin compared with the mean power over the baseline epoch for that frequency, $TFR=\frac{\text{P}\left(\text{t},\text{f}\right)-{\text{P}}_{\text{baseline}}\left(\text{f}\right)}{{\text{P}}_{\text{baseline}}\left(\text{f}\right)}.100$ where *P(t,f)* = power at time *t* and frequency *f* and P_baseline_(f) = mean activity at frequency *f* over the baseline epoch.

This procedure yields TFRs containing phase-locked as well as non-phase-locked responses.

#### Induced time-frequency representation (I_TFR_):

In studying oscillatory epileptic spike, it is possible that any change in oscillatory activity (especially higher frequency) that is related to an IES is time-locked to this IES but not necessarily phase-locked. The reason is that oscillations are ongoing phenomena that also exist in the absence of any IES. As a result, the phase of the oscillation at the time of occurrence of an IES is variable. In order to specifically assess the non-phase locked activity of IES (called induced activity), we subtracted the time-frequency representation of patient's averaged trials from the Global Time-Frequency Representation to create averages of the non-phase-locked spectral power only (see Figure 1 for detailed analysis block diagram).

##### Statistical analysis

The probability that a power differs significantly from the average power during the baseline interval was investigated. Two-sided bootstrap testing were performed on the trials (Davidson, 1999). z0 and the test statistics z^*^ for a given bootstrap sample were computed as

$$\begin{array}{l} z0=\frac{{ȳ}_{2}-{ȳ}_{1}}{\sqrt{\frac{\sigma_{y_{2}}^{2}}{n}+\frac{\sigma_{y_{2}}^{2}}{n}}},z^{*}=\frac{{ȳ}_{2}^{*}-{ȳ}_{1}^{*}-\left({ȳ}_{2}-{ȳ}_{1}\right)}{\sqrt{\frac{\sigma_{y_{2}}^{2}}{n}+\frac{\sigma_{y_{2}}^{2}}{n}}} \end{array}$$

Where,

$$\begin{array}{l} {ȳ}_{1}=\frac{1}{n}\underset{trials}{\sum }{\bar{P}}_{baseline,i}\\ {ȳ}_{2}=\frac{1}{n}\underset{trials}{\sum }P_{i} \end{array}$$

Here P denotes the power, n the number of trials. An asterisk denotes the value of a bootstrap sample. R bootstrap samples were computed. This computation was performed for each sampling point in time-frequency space. The *p* value was approximated from the number of bootstrap samples where ${\text{z*}}^{\text{2}}>{\text{z}}_{0}^{2}:p=\left(1+\#\left\{{\text{z*}}^{\text{2}}>{\text{z}}_{0}^{2}\right\}\right)/\left(\text{R}+1\right)$.

To reduce the false positive rate (FPR), correction for multiple testing was performed using the method of Simes (1986). It is applied to each IES epoch which belongs to one frequency bin. This means that each channel and each frequency bin was treated as an independent measurement, whereas the statistical tests over the time series within one frequency bin were treated as multiple measurements. This approach was suggested by Auranen (2002). The reasoning behind it is that, it is difficult to define a rule for dependencies between channels. Each montage and each physiological phenomenon leads to different dependencies between the measurement channels. It was assumed that the activity which we were interested in is based on oscillatory phenomena, which were likely to be confounded to defined frequency bands. To assess the strength of the observed effects and for FPR correction, all *p* values of one frequency bin and channel were sorted in ascending order (p_*i*_, *i* = *1,…,N*). The maximum index *m* in the sorted array for which p_*i*_ < α^*^i/N was determined. All values with *i* < *m* were accepted as significant detection. The significance level α is set to 0.05.

#### Control conditions

##### Random triggers

To reach the specificity of IES time-frequency representations, all the same 3-step procedure of time frequency analysis was performed with control random triggers.

Control random segments (*n* = 197), lasting 2000 ms around the random triggers, were analyzed. They were selected during similar period and background activities during which the effects of IES were analyzed. The 1000 ms before each EEG epoch and the epoch (lasting 2000 ms) did not contain any IES. T0 was defined by random trigger time which corresponds to the central point of the epoch (−1000 ms, +1000 ms).

The TFR procedure described above was performed using the baseline segments lasting 400 ms (−1000 to −600 ms before T0).”

##### BECTS spikes simulation

In order to reproduce the BECTS EEG data, one equivalent current dipole was fitted, located in the left central sulcus and characterized by an oblique orientation toward the mid-line, EEG data was generated using a spherical four-shell head model (Berg and Scherg, 1994; BESA® Dipole Simulator) with the following parameters.

- Radius of the head model 85 mm
- Thickness of layers (mm):
  ◦ Scalp 6 mm
  ◦ Bone 7 mm
  ◦ CSF 1 mm
- Conductivities (mho/m):
  ◦ Scalp 0.33
  ◦ Bone 0.0042
  ◦ CSF 1.0
  ◦ Brain 0.33

All the TFR procedure has been applied to the simulated IES.

## Results

Eight male patients were included in the study. On HD EEG recording (64 electrodes), unilateral IES were selected in 6 patients. They were bilateral and independent in 2 other patients. This resulted in a total of 10 groups of IES.

### Clinical data (Table 1)

According to the inclusion criteria, all patients presented sensori-motor seizures with or without secondary generalization, regardless of whether IES were unilateral or bilateral. Five patients were taking antiepileptic drugs at the time of the recording. Two patients were not seizure-free. Four children had attention disorder and language impairment with no global deficit on the Wechsler Intelligence Scale for Children (WISC) IV test.

**Table 1:** **Clinical data for the 8 patients with BECTS**.

**Patients**	**Age at diagnosis of BECTS (years)**	**Clinical features of seizures**	Seizure-free^*^	**Antiepileptic drugs**	**Standard EEG**	**Neuropsychological data**
1	5	GTCs	YES	–	Left CTs	Attention deficit
2	5	GTCs	YES	VPS	Right CTs	Attention deficit, Language impairment
3	7	PS	YES	–	Right and left CTs	Normal
4	9	PS	NO	–	Right CTs	Normal
5	8	PS	YES	LVM-VPS	Left CTs	Normal
6	9	PS	YES	VP	Right CTs	Attention deficit
7	5	PS	NO	VPS-OXC	Right & left CTs	Attention deficit
8	6	PS	YES	VPS-OXC	Left CTs	Normal

GTCs, Generalized Tonic-Clonic seizures; PS, Partial Sensorimotor seizures; VPS, Sodium Valproate; LVM, Levetiracetam; OXC: Oxcarbamazepine; CTs, Centrotemporal spikes;

* *at the time of HD EEG*.

### Source localization (Figure 2)

The Source localization of the IES was performed in order to further assess the homogeneity of patients' population.

**Figure 2:**
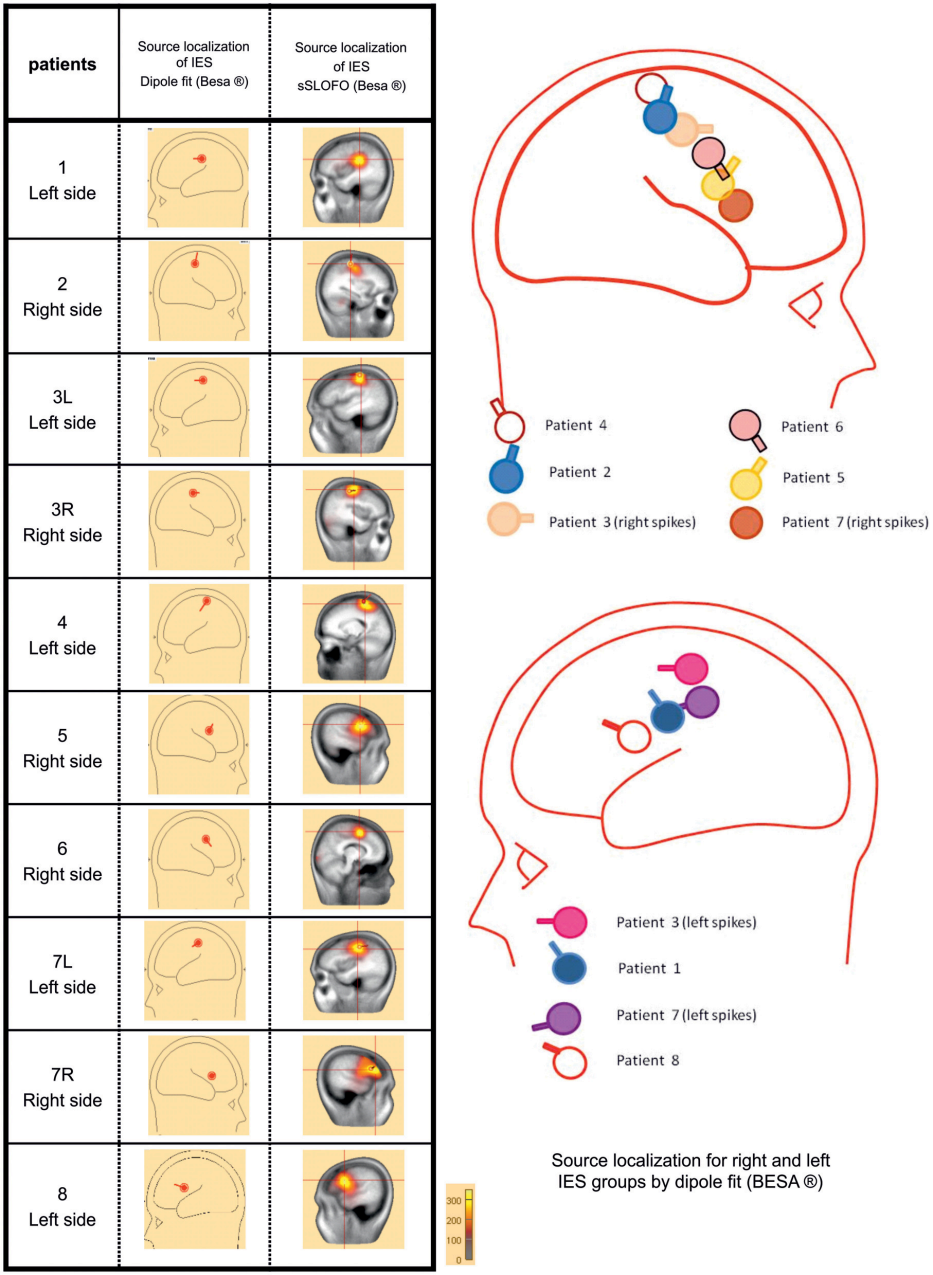
**Source localization using the dipole fitting method and the distributed methods for each group of IES**. In all children, source localization, whatever the method used confirmed the origin of the IES, along the central sulcus with a tangential dipole orientation and an anterior positivity consistent with a precentral origin.

In all children, interictal source localization, using a dipole (Dipole fit) or distributed method (SSLOFO), confirmed the origin of the IES along the central sulcus (Ishitobi et al., 2005). The localization of the dipole at the first negative deflexion, and its tangential orientation with an anterior positivity was consistent with the expected precentral origin in BECTS patients (Ishitobi et al., 2005) (Figure 2). The patients' population was therefore considered to be clinically and electrophysiologically homogeneous, allowing further analysis.

### Time-frequency analysis

The first step consisted of computing local changes in synchronization surrounding the IES for all frequency bands. Changes in synchronization occurring simultaneously around the IES, in areas distant to the epileptogenic zone were then analyzed.

#### Local synchronization changes (Table 2, Figures 3, 4)

For all groups of IES, time-frequency analysis demonstrated statistically significant changes (*p* < 0.0002) (±400 ms around the IES) compared to the 2 reference periods (−600, −1000 ms and −3000, −1000 ms before IES), for both G_TFR_ and I_TFR_.

**Table 2:** **Results of global and induced time-frequency responses for all IES groups, simultaneously with IES and locally or at a distance around IES**.

***Patients***	***Simultaneously with IES***		***Around IES***	
	**4—50 HZ**	**> 50 HZ**	**Local synchronization 4–50 Hz band**	**Distant synchronization 4–10 Hz band**
1	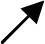 *G* NS *TFR: 70%, I :59%*	NS	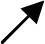 *GTFR: 42%, ITFR: 35%*	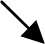 AF3-FP1-F1-F3-F5-AF8-F4-F6-C6-P2-P4-TP8-TP10-C1-C7-Fz-Pz-POz
2	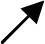 *GTFR: 100%, ITFR: 67%*	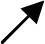 *GTFR: 28%, ITFR: 11%*	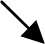 *GTFR: 95%, ITFR: 84%*	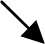 ^*^
3L	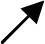 *GTFR: 75%, ITFR: 12%*	NS	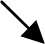 *GTFR: 20%, ITFR: 3%*	NS
3R	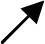 *GTFR: 72%, ITFR: 25%*	NS	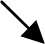 *GTFR: 10%, ITFR: 4%*	NS
4	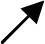 *GTFR: 100%, ITFR: 100%*	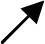 *GTFR: 48%, ITFR: 45%*	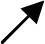 *GTFR: 100 %, ITFR: 100 %*	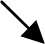 Fz-FP2-AF8-FC3
5	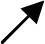 *GTFR: 89 %, ITFR: 33 %*	NS	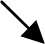 *GTFR: 16 %, ITFR: 8 %*	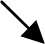 AF8-FC1-FC5-FT7-F7-TP9-TP7-CP1-CP2-P8-PO4-PO8
6	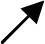 *GTFR: 100%, ITFR: 100%*	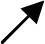 *GTFR: 61%, ITFR: 61%*	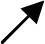 *GTFR: 91%, ITFR: 91%*	NS
7L	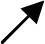 *GTFR: 78%, ITFR: 34%*	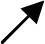 *GTFR: 11%, ITFR: 9%*	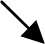 *GTFR: 27%, ITFR: 14%*	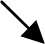 AF7-FPz-AFz-FPz-FP2-AF4-AF8
7R	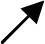 *GTFR: 100%, ITFR: 73%*	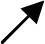 *GTFR: 42%, ITFR: 41%*	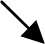 *GTFR: 53%, ITFR: 44%*	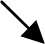 AF7-FP1-FPz-FP2
8	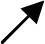 *GTFR: 97%, ITFR: 30%*	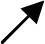 *GTFR: 2%, ITFR: 2%*	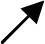 *GTFR: 53%, ITFR: 37%*	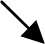 AF7-FP1FPz-FP2-AF8-F7-F8-FT8

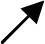
 Increase of power signal analyzed by time-frequency method (baseline [−1000; −600 ms] (p < 0.0002)

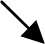
 Decrease of power signal analyzed by time-frequency method (baseline [−1000 ms; −600 ms] (p < 0.0002)

*NS, Not Significant; G_TFR_, Global Time-frequency Response; I_TFR_, Induced Time-frequency Response; x %, number of electrodes involved in changes of synchronization; 3L, left IES for patient 3; 3R, right IES for patient 3; 7L, left IES for patient 7; 7R,right IES for patient 7*.

* Electrodes involved in decreased distant synchronization for patient 2: AF3-AF7-FP1-F1-F3-F5-F7FC1-FC5-FC3-FT7-C1-C3-C5-T7-CP1-Cp3-Cp5-TP7-TP9-P3-P5-P7-O1-PO3-PO7-FPz-AFz-Fz-FCz-Cz-CPz-Pz-POz-Oz-FP2-AF4-AF8-F2-F4-F6-F8-FC2-FC4-FC6-FT8-C6-T8- Cp6-TP8-P6-O2-PO4

**Figure 3:**
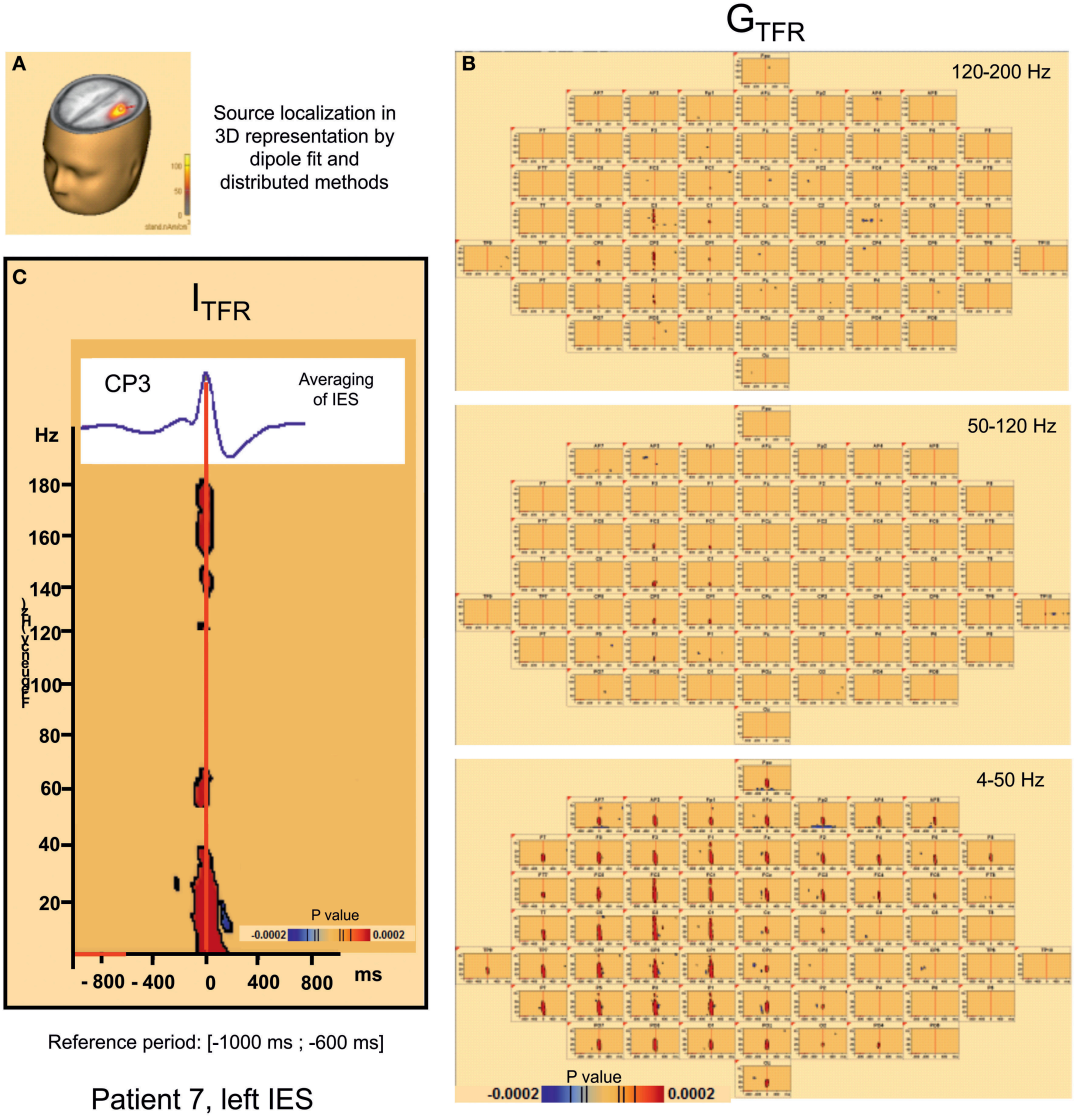
**Hypersynchronisation occurring simultaneously with the IES in the 4–200 Hz band in patient 7 (left IES) in Global (G_**TFR**_) and Induced responses (I_**TFR**_). (A)** Source localization in dipole fit and sSLOFO in a 3D representation for the patient 7. **(B)** Significant Statistical results (*p* < 0.0002) of time frequency analysis (induced activity) for frequencies between 4 and 50 Hz, 50 and 120 Hz, and 120–200 Hz. **(C)** On the top, result of the averaging of the IES for the patient 7. By time frequency analysis, Island of HFOs are simultaneously extracted with the IES, in induced activity (I_TRF_).

**Figure 4:**
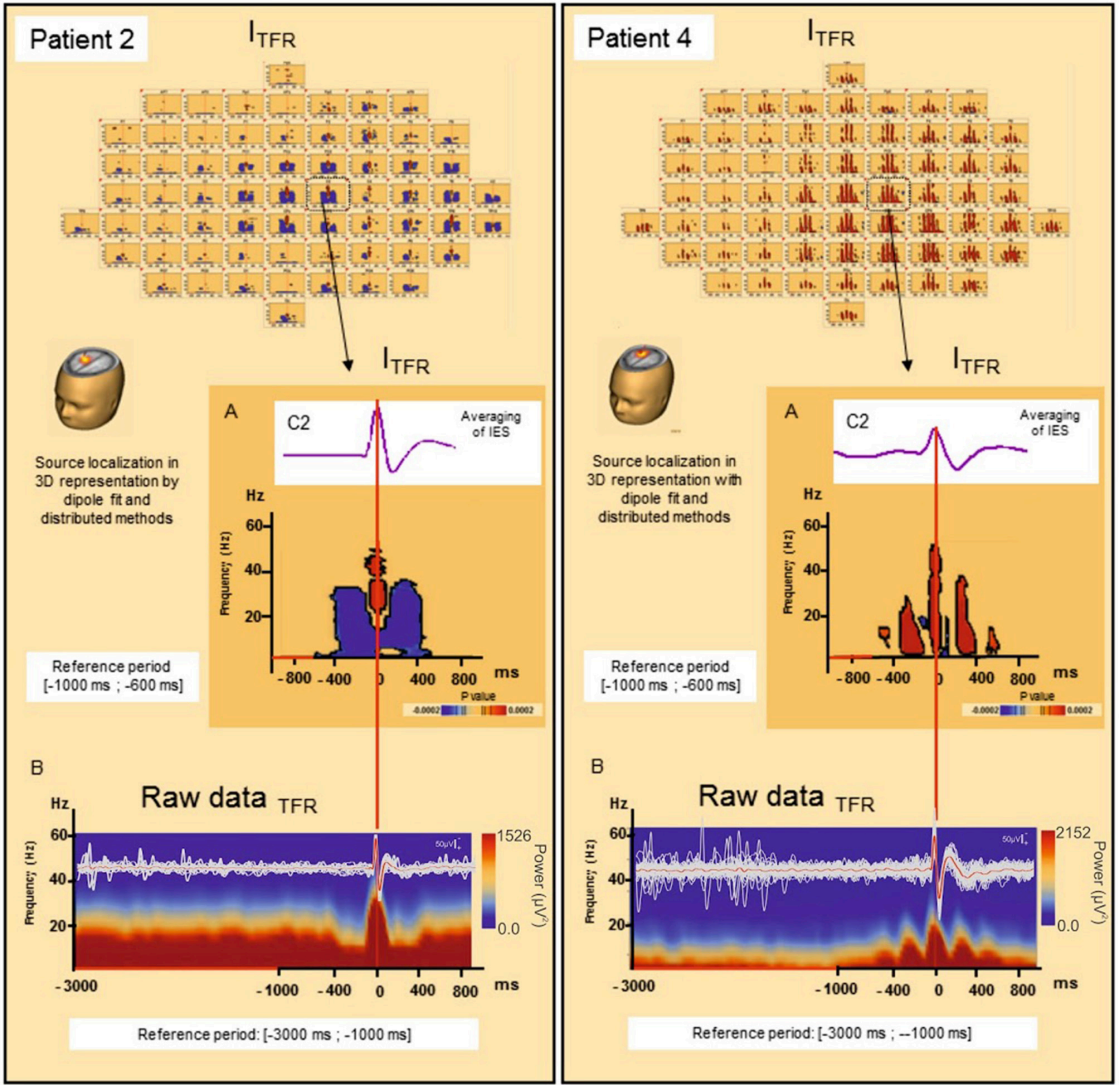
**Induced local changes in synchronization around the IES, examples of patients 2 and 4. (A)** On the top, results of the averaging of the selected IES. Bellow, Significant statistic (*p* < 0.0002) results of time frequency analysis in regard of the electrode C2 for patient 2 (on the left) and patient 4 (on the right) (reference period [−1000; −600 ms]). **(B)** Raw data of time frequency analysis for the patient 2 (on the left) and 4 (on the right) (reference period: [−3000; −1000 ms]). For patient 2, I_TFR_ showed mirrored desynchronization around T0 for frequencies range from 4 to 50 Hz independently of the baseline considered [−1000; −600 ms] **(A)** or [−3000 ms; −1000 ms] **(B)**. This desynchronization was localized nearly of the epileptogenic zone. For patient 4, a progressive increase in synchronization mirrored around the IES, in the same frequencies were found [baseline [−1000; −600 ms] (A) or [−3000 ms; −1000 ms] **(B)**]. T0: define by the peak of the first negative deflexion of the IES.

##### Hypersynchronization occurring simultaneously with the IES (see Table 2, Figure 3)

Independently of the baseline considered, signal power increased significantly (*p*_corrected_ < 0.0002) in G_TFR_ between 4 and 50 Hz (Figure 3). This hypersynchronization involved 88% of electrodes [45 to 64 (70–100%) electrodes] (Table 2). Similar results were observed for I_TFR_ (*p*_corrected_ < 0.0002) (Figure 3), but with a narrower spatial extension around the epileptogenic zone, with only 53% of electrodes involved [8 to 64 (12–100%) electrodes] (Table 2).

At frequencies higher than 50 Hz, a significant (*p*_corrected_ < 0.0002) increase in signal power was observed for 6 of the 10 groups of IES. The spatial extension included 32% (1 to 39 electrodes) and 28% (1 to 39 electrodes) of the electrodes in G_TFR_ and I_TFR_ respectively (Table 2). These significant high-frequency synchronizations occurred concomitantly and continuously with hypersynchronization of lower frequencies.

##### Time-frequency changes surrounding the IES (Table 2, Figure 4)

Independently of the baseline considered, G_TFR_ were significantly modified, between 4 and 50 Hz, in the −400 to +400 ms window around the hypersynchronization of the IES (T0) for all groups of IES. No changes surrounding T0 were observed in the frequency domain higher than 50 Hz.

Two different patterns of synchronization changes were observed in the time-frequency domain.

*Pattern 1:* For 6 of the 10 groups of IES, time-frequency changes consisted of a significant (*p* < 0.0002) decrease in the power of frequencies below 50 Hz before (−400,−100 ms) and after (+100, +400 ms) the IES, likely corresponding to a decrease in synchronization preceding and following hypersynchronization of the IES, i.e., a kind of mirror desynchronization surrounding the IES (Figure 4). In G_TFR_, this desynchronization involved 37% of electrodes (10 to 95% of electrodes). Similar results were observed for I_TFR_, but over a more limited area (26% of electrodes, 3–84%) adjacent to the epileptogenic zone (Table 2).
*Pattern 2*: Instead of the desynchronization described in pattern 1, a significant (*p* < 0.0002) oscillation in hypersynchronization was observed in the same time window (−400, +400 ms) surrounding hypersynchronization of the IES (−100, +100 ms) for 4 of the 10 groups of IES (Figure 3). The spatial extension included 71% (42 to 100%) and 66% (35 to 100%) of electrodes in G_TFR_ and I_TFR_, respectively (Table 2).

It should be stressed that neither pattern 1 nor pattern 2 was correlated with any changes in HD EEG raw activity, including the slow waves preceding or following the IES (see Figure S2).

TFRs were not affected by the use of different baselines (Figure 4).

#### Distant synchronization changes surrounding the IES (Table 2, Figure 5)

In 7 of the 10 groups of IES, a significant decrease (*p* < 0.0002) in signal power was observed for G_TFR_ and I_TFR_ at frequencies below 10 Hz in areas distant from the IES onset zone. This distant desynchronization occurred during the same time window (−400, +400 ms) during which the synchronization power was either decreased (pattern 1) or oscillatory (pattern 2) in the epileptogenic zone (Table 2) (Figure 4). These desynchronizations in low-frequency bands occurred in frontal (*n* = 7) and/or temporal (*n* = 4) and/or occipital (*n* = 2) areas are highly suggestive of repetitive and transient desynchronizations distant from the epileptogenic zone of the IES.

**Figure 5:**
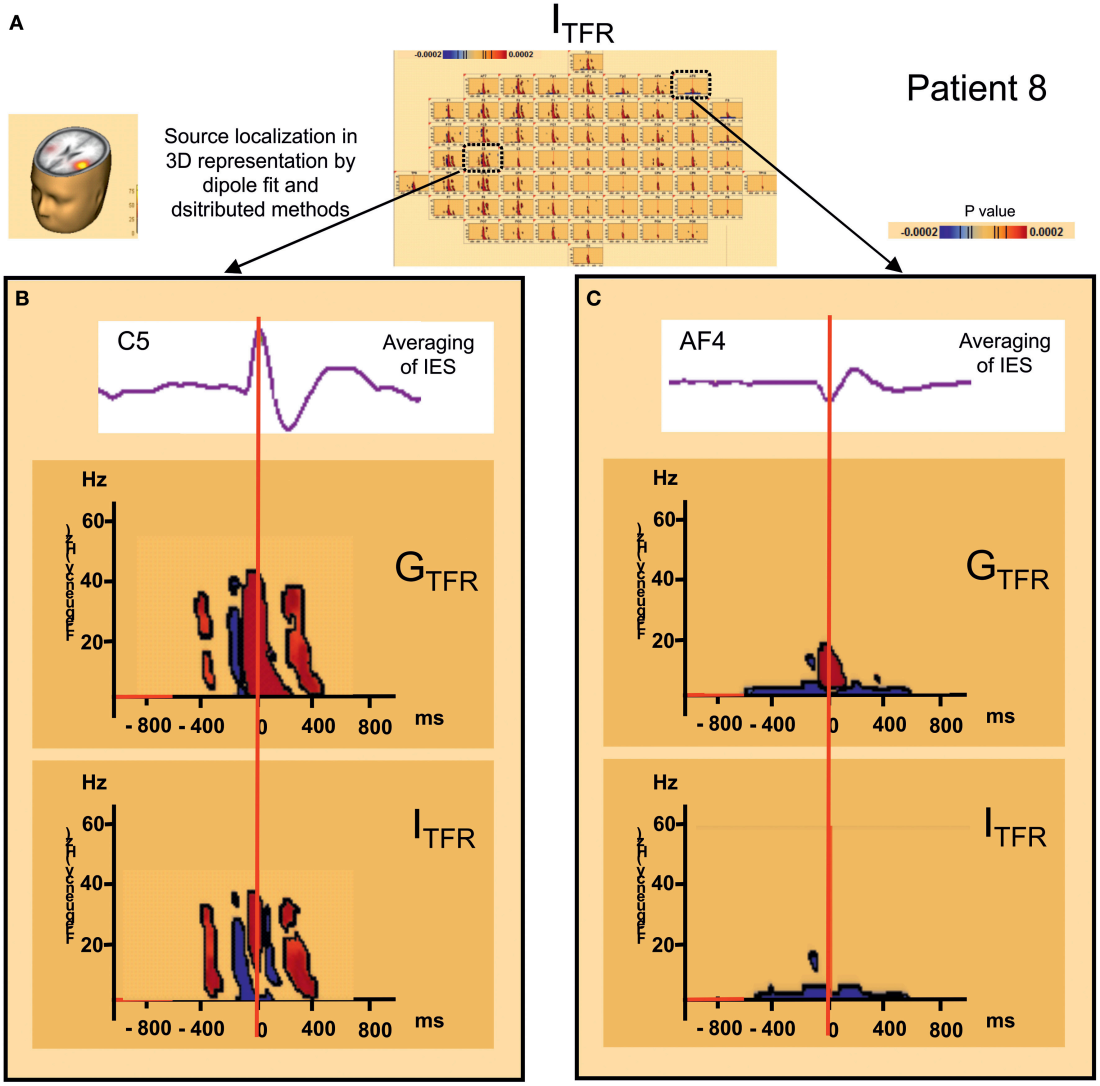
**Distant changes in synchronization (G_**TFR**_ and I_**TFR**_) around IES. (A)** Source localization and dipole fit and SSLOFO in a 3D representation for the patient 8. **(B)** Statistical significant results (*p* < 0.0002) of time frequency analysis [induced (I_TFR_) and global (G_TFR_) time frequency results] in regard of the epileptogenic focus (electrode C5). **(C)** Statistical significant results (*p* < 0.0002) of time frequency analysis [induced (I_TFR_) and global (G_TFR_) time frequency results] in regard of the frontal area (electrode AF4). In I_TFR_ and G_TFR_ distant desynchronizations were observed, distant to the epileptogenic zone, notably in fronto-temporal areas, involving low frequency bands (bellow 10 Hz) **(C)** in the same time window as local dysregulation of synchrony **(B)** (Figure 3) [−400 ms; +400 ms].

Like for TFRs observed in the epileptogenic zone, TFRs in distant areas were not affected by the different baselines (Figure 4).

#### Time-frequency changes in control conditions (Figures 6, 7)

In order to evaluate the specificity of low-frequency activity, exactly the same time-frequency analysis that we have done for the IES was performed but using the random triggers, out of IES. Time frequency analysis EEG activities related to the random trigger do not produce any statistically significant effects around the trigger, nor were hypersynchronization or desynchronization observed in either the epileptic zone or in the distant areas.

**Figure 6:**
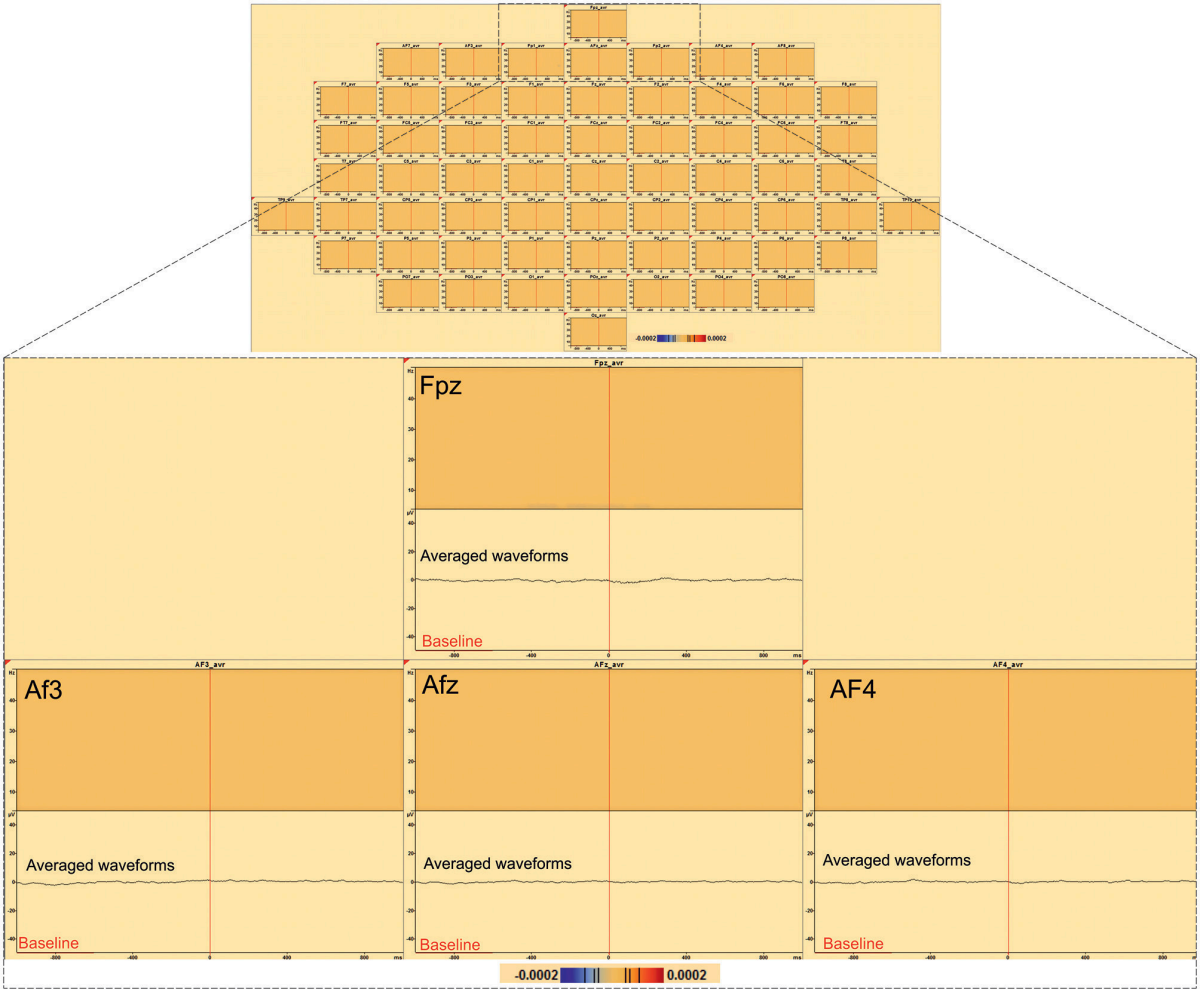
**Time-frequency analysis of 197 random triggers out of IES in patient 8**. No significant statistical (*p* < 0.0002, corrected *p*-value) changes in random triggers out of IES were found by time frequency analysis (baseline [−1000; −600 ms]).

**Figure 7:**
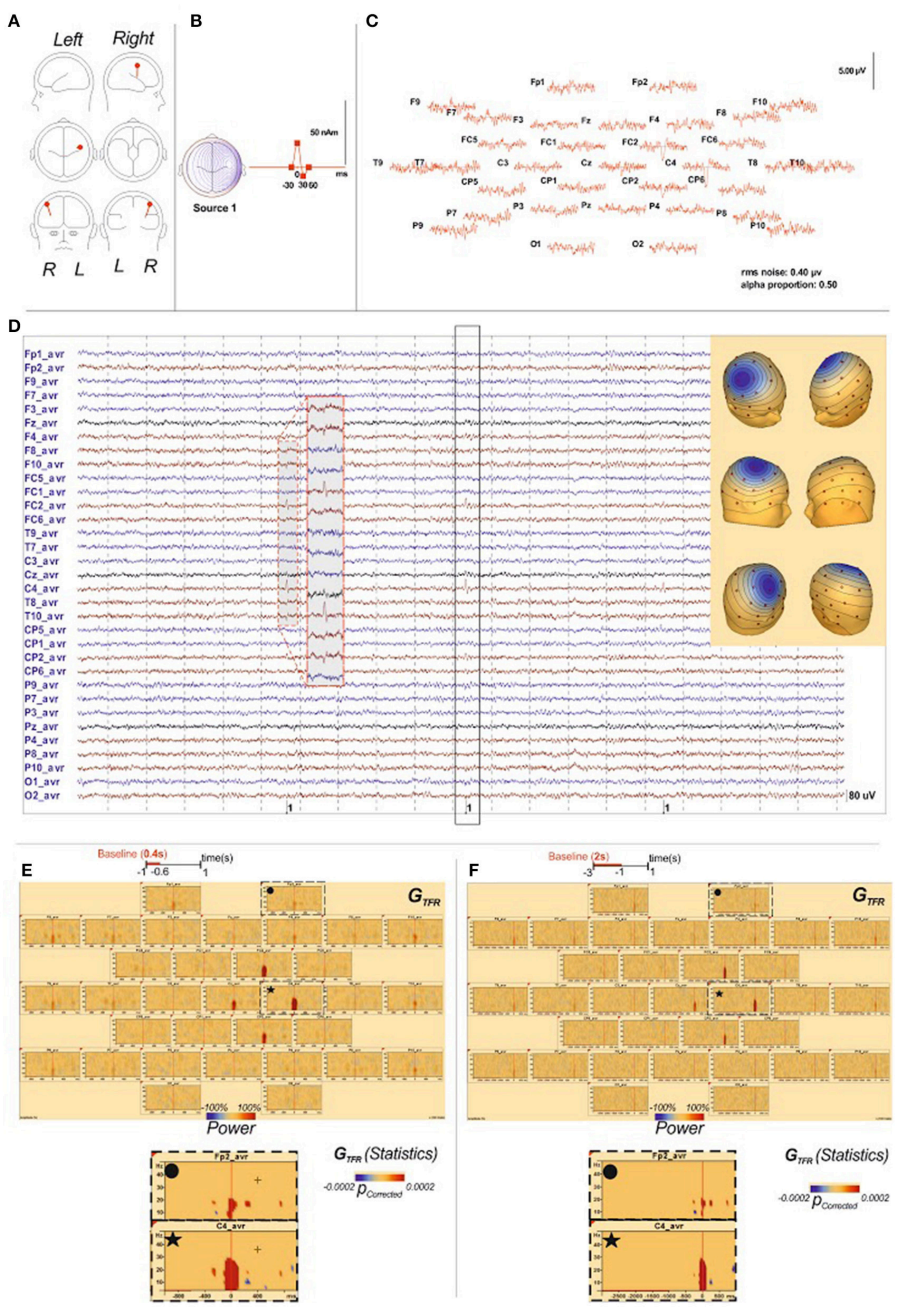
**Time-frequency analysis of IES simulation. (A)** The source localization by dipole fit method identified an origin of the simulated IES in the right central sulcus, like described in BECTS (Ishitobi et al., 2005). Locations, orientations **(A)** and wave shapes **(B)** of the 1 dipole source, “the model sources,” used to simulate the EEG data. T0 is denoted by the vertical mark on the time base. **(B)** These topographies reproduced properties expected for the scalp-recorded IES field, including central negativities, with corresponding positivities at lateral sites. **(C)** The simulated scalp IES in average reference format. The solid lines indicate the noise-free wave forms generated by the dipole sources indicated in Panel **A**. **(C)** indicate the signal + noise wave forms that were analyzed. **(D)** raw data of the simulated EEG and IES. Scalp potentials (33-channel montage) were simulated from dipoles (Berg, 2006) in order to correspond to field produced by the generator within right central sulcus. **(E)**: G_TFR_ of the simulated IES results with the [−1000 ms; −600 ms] baseline period: G_TFR_ of the simulated IES demonstrated significant changes between 4 and 30 Hz in the right central sulcus simultaneously with the IES but no change around the IES in regard of the epileptogenic zone or in distant area. **(F)**: G_TFR_ of the simulated IES results with the [−1000 ms; −600 ms] baseline period: Like for the [−1000 ms; −600 ms] baseline period, G_TFR_ of the simulated IES demonstrated significant changes between 4 and 30 Hz in the right central sulcus simultaneously with the IES but no change around the IES in regard of the epileptogenic zone or in distant area.

The TFR analysis performed on simulated IES returned isolated local hypersynchronization in G_TFR_ in 4–30 Hz frequencies. Neither local nor distant changes surrounding IES were identified whatever the baseline period (Figures 5E,F, Figure S1).

## Discussion

Time-frequency analysis applied to HD EEG in a homogeneous population of male patients with BECTS demonstrated neuronal synchronization changes surrounding IES. Before and after the well-known hypersynchronization concomitant with the IES (±400 ms) there were: (i) two patterns of dysregulation in the epileptogenic zone and (ii) distant desynchronization, involving low-frequency bands in frontal, temporal, and occipital networks. These dysregulations might be involved in the mechanisms that propel neurons to synchronize and may play a role in the cognitive deficits observed in BECTS patients (Vannest et al., 2015).

### Methodological considerations

Because in BECTS, sources are modeled by one tangential source (Pataraia et al., 2008; Kakisaka et al., 2011), we analyse EEG potentials in the sensor space. The analysis in the sensor space might have led to overlap between the information of adjacent electrodes due to volume conduction effect which in turn may lead to spurious connectivity among neighboring channels. Surface Laplacian, might have been an alternative since it have the effect of reducing the effective volume, thereby improving spatial resolution; as discussed in Nunez and Srinivasan (2006), potentials and surface Laplacians are sensitive to different spatial band-widths of the source distribution. Thus, surface Laplacians serve to complement (but not replace) EEG potentials (i.e., the surface Laplacian emphasizes certain types of source activity—More sensitive to radial than tangential dipoles, thus sources in sulci will be minimized—reduces the sensitivity of the EEG to sulci). In this paper we focused on the dynamics of neuronal networks surrounding IES which might improve our knowledge of the mechanisms that drive neurons to the hypersynchronization in BECTS. In our recently published connectivity study of IES in BECTS (Adebimpe et al., 2016), to identify the average location of interictal spike sources we used the exact Low Resolution Electromagnetic Tomography (eLORETA) method. Both approach returns similar results concerning the sources and the interactions of the IES with the frontal areas (Adebimpe et al., 2016)

*Simultaneously with the IES*, a significant broad band increase in the power spectrum was observed, including frequencies higher than 50 Hz.

To exclude the presence of false synchronizations caused by filtering of sharp transients (Bénar et al., 2010; Amiri et al., 2016), induced activities were extracted from global changes in synchronization. By means of this original approach, we showed that IES in BECTS are associated with a significant increase in high-frequency power (50–200 Hz), co-occurring with IES.

These increases could reflect, partially, high frequency oscillations (HFOs) widely recognized as a marker of epileptic tissue in partial refractory epilepsy, (Jacobs et al., 2011, 2012, 2016; Jefferys et al., 2012) but also observed in idiopathic partial epilepsies (Kobayashi et al., 2011; van Klink et al., 2016).

In our study, high frequencies power increases were more localized than the increases in lower frequencies, suggesting that they occurred in more restricted centro-temporal areas. Despite a potential volume conduction effect on the extension of HFO and their localization value, our results are in accordance with previous studies which showed, independently of the type of epilepsy, that spikes with HFOs are closely linked to the epileptogenic zone (Jacobs et al., 2009a). This remark also applied to the volume conduction effect on low frequency bands distant desynchronizations. They were not observed in the epileptogenic zone but only in restricted frontal areas and also unilateral. Altogether, this suggests that the volume conduction effect does not impact so much the localization in BECTS. However, the evaluation of the extension of the synchronization or desynchronization deserves further studies. One issue which could help to minimize this effect (Kayser and Tenke, 2006; Srinivasan et al., 2007) would be, to apply a spatial high-pass filter or other spatial transform such as current-source-density, to reject zero-phase lag synchronizations (König et al., 1995; Rajagovindan and Ding, 2008; Vicente et al., 2008), to apply independent components analysis, which calculates unique generators of variance in the cortex (Makeig et al., 1997) or to estimate the cortical sources using beamforming (e.g., LCMV).

*Hypersynchronization was preceded and followed by complex dysregulation of synchronization* occurring within the epileptogenic zone and characterized by 2 specific patterns between 4 and 50 Hz: (i) a decrease in power frequencies and (ii) oscillation of power frequencies.

As this dysregulation consisted of 2 different patterns and was not affected by the use of different reference periods, it is unlikely to be due to a signal processing artifact or the resulting effect of a preceding masked IES not visualized on raw data. Moreover, Keller and collaborators found similar results in ECoG (Keller et al., 2010).

In the present study, we focused on changes in synchronization surrounding the IES, especially those changes preceding the IES. The references periods (−1000; −600 ms or −3000; −1000 ms) therefore had to be selected in the same activation state, in the immediate temporal environment of the peak and away from the time window of interest (−400; +400 ms). This requirement may explain some of the differences observed between our study and other published studies, in which a single reference period was chosen situated at a distance from the peaks (Jacobs et al., 2011) or constructed as representing an average brain activation state (Kobayashi et al., 2009). In these previous studies, the authors only identified desynchronizations after the IES (Kobayashi et al., 2009; Jacobs et al., 2011) and/or an inconstant hypersynchronization occurring before the IES that the authors characterized as HFOs (Kobayashi et al., 2009; Ren et al., 2015).

The mechanisms involved in these complex dysregulations have not yet been elucidated. They cannot be explained by synaptic interactions, gap junctions or ephaptic conduction involved in intrinsic membrane oscillations and responsible for the generation of HFOs (Jefferys et al., 2012), as they started only a few tenths of milliseconds before synchronization of the IES. Similarly, mechanisms proposed for the emergence of the IES are unlikely to contribute to the observed dysregulations. Although the Potential Depolarization Shift (PDS), the hallmark of the IES (Ayala, 1983), is much longer than the depolarization observed with normal excitatory post-synaptic potentials, the progressive recruitment of excitatory inputs that trigger the IES start only several tenths of milliseconds before onset of the IES, which does not correspond to the time window of 400 ms observed in our study. Other mechanisms, such as gap junction and calcium waves, notably involved in the initiation and propagation of synchronization to neighboring neurons, have been reported to be triggered simultaneously with the IES (Jefferys et al., 2012). Finally, the hyperpolarization following PDS, corresponding to the slow component of the spike wave discharge in EEG (Ayala, 1983; Neckelmann et al., 2000) cannot explain the desynchronization observed after the IES, as, in line with previous reports, desynchronization was observed regardless of whether or not the slow wave was present and therefore does not necessarily reflect the degree of post-spike depression (Jacobs et al., 2011).

It may be more relevant to consider our results at a mesoscopic/macroscopic level. The dynamics of the assembly of neurons in the epileptic network involved in the emergence of the IES are more complex, more heterogeneous and more variable than initially thought. Using multi-unit activity analysis in refractory epilepsy, Keller and collaborators observed a marked variability of cellular activation pattern (decrease or increase of neuronal activity) within the seizure onset zone, concomitantly to the IES (Keller et al., 2010). Moreover, activity changes were observed for some clusters of neurons at longer interval (400 ms) before the IES (Keller et al., 2010; Alvarado-Rojas et al., 2013). Similarly, using the Fast Optical Signal (FOS) technique synchronized with ECoG in epileptic rats, we have shown changes in cellular conformation in the same time range (Manoochehri et al., 2017) suggesting cellular activations occurring well-before the IES. The two types of dysregulation observed in the present study are in line with these results and represent, at a macroscopic level, the variability of the strategy of neuronal assemblies to reach the freezing point beyond which recruitment of synaptic inputs will trigger the regenerative currents of the PDS and IES (Keller et al., 2010; Alvarado-Rojas et al., 2013; Manoochehri et al., 2017). Similarly, using a graph-theoretical approach, increases in network clustering around the IES, in both symptomatic partial epilepsy (Ibrahim et al., 2014) and BECTS (Adebimpe et al., 2015a,b) suggest a more complex reorganization of the network in the epileptogenic zone, which can result in changes in the synchronization dynamics, as monitored by time-frequency analysis of scalp HD EEG.

### Desynchronization in low-frequency bands, distant to the epileptogenic zone

In parallel to local desynchronization, our study demonstrates desynchronization in low-frequency bands, distant to the epileptogenic zone.

Like local reorganization of the neuronal networks, distant inward interactions are also likely to modify the functional environment inside the epileptic zone in which IES are triggered and the functional connectivity of the epileptic zone toward other areas (Adebimpe et al., 2015a,b, 2016). By extrapolating from the features described in seizures, in which desynchronizations or hypersynchronizations are observed several minutes or hours before the seizures, the dysregulation of synchronization observed in the present study would modify the input complexity and functionality of epileptogenic brain regions, creating an idle population of neurons that may be more susceptible to recruitment into IES (Le Van Quyen et al., 2005; Aarabi et al., 2008). Similarly, the previously described hemodynamic changes starting several seconds before the IES (Jacobs et al., 2009b; Osharina et al., 2010) are likely to modify the functional environment of the network around the IES.

However, the effect observed in distant areas is highly specific to the interictal spikes and are only observed ±500ms around the peak of the spikes concomitantly to the alternation of desynchronization-synchronization-desynchronization observed in the epileptic zone. In line with these results, by combining the EEG source imaging and the time varying effective connectivity method, stronger directional connections from the epileptic zone to the frontal regions were observed during interictal spikes in BECTS patients (Adebimpe et al., 2016) suggesting that benign epileptic network may be disrupted by IES (Adebimpe et al., 2015a,b).

It has long been suspected that IES contribute to cognitive and behavioral deficits (15–30% of BECTS children), but the underlying physiological mechanisms are still poorly understood (Vannest et al., 2015). In BECTS, IES are associated with transient cognitive impairment (TCI) (Aarts et al., 1984; Fonseca et al., 2007), which starts several hundreds of milliseconds before the spikes (Van Bogaert et al., 2012). Reorganization of the networks in the epileptic focus and in other functionally connected areas might participate in TCI (Verrotti et al., 2014). In support of this hypothesis, connectivity analysis using EEG or fMRI has demonstrated deactivation associated with IES in widespread cortical areas involved in cognitive processing, including the frontal, temporal and occipital cortex (Besenyei et al., 2012; Adebimpe et al., 2015a; Xiao et al., 2016), as well as areas involved in the Default Mode Network (DMN) (Cataldi et al., 2013; Fahoum et al., 2013; Adebimpe et al., 2015b). IES would therefore repetitively and transiently disrupt the functionality of different networks involved in cognitive processing (Besseling et al., 2013; Verrotti et al., 2014). Although our study does not allow a straight full demonstration of the relationship between distant desynchronization and cognitive disorders, the concomitancy of desynchronization in distant areas constitutes one possible substrate for the functional disruption and network reorganization leading to TCI. Further prospective studies including neuropsychological tests performed at the time of the EEG are mandatory to more specifically evaluate the potential underlying mechanisms explaining the cognitive consequences of IES. Our results, notably in distant areas, constitute just the beginning of unraveling the nature of cognitive deficits in this population.

## Conclusion

In the present study, we show that large-scale network changes also precede the IES, supporting the concept that epileptic dynamics cannot be viewed in isolation, but must be interpreted in the context of a dynamic system of interregional communication within larger networks.

In the epileptogenic zone, complex changes both shortly preceding and following IES, illustrated the complexity of the underlying mechanisms of IES generation.

Changes in desynchronization are also observed in distant zones from the epileptogenic area both shortly before and after IES, suggesting that neuronal reorganization is under the influence of a larger embedded network, which may play a causal role in the expression of the IES.

In concordance with other studies, these findings observed in male patients with BECTS are likely to be generalizable across different gender or other underlying epileptogenic syndromes and locations of epileptic foci.

TFRs on scalp HD EEG would open new perspectives about the understanding of the mechanisms of IES generation and, through future studies, the causal role of local large-scale networks on changes to cognitive deficits.

## Author contributions

Conceived and designed the experiments: EB, MM, PB, and FW. Performed the experiments: EB and MM. Contributed reagents/materials/analysis tool: EB, MM, and FW. Wrote the paper: EB, MM, and FW. Read and accepted the manuscript: EB, MM, and PB, and FW.

### Conflict of interest statement

The authors declare that the research was conducted in the absence of any commercial or financial relationships that could be construed as a potential conflict of interest.

## Supplementary material

Figure S1 **Impact of varying slow wave amplitude on time-frequency analysis. (A)** Simulated IES dipole in the right central sulcus, Locations, orientations. **(B)** Simulated IES with increasing slow wave amplitudes (0, 5, 10, 15, 20 nAm). **(C)** The simulated scalp IES in average reference format. The solid lines indicate the signal + noise wave forms generated by the dipole sources indicated in **(A)**. **(D)** Butterfly plot of the simulated spikes, Scalp potentials (33-channel montage) were simulated from dipole in order to correspond to field produced by the generator within right central sulcus. *(Up-left)* superimposing all channels. **(E)** The absolute power of time-frequency analysis is displayed. The x-axis shows the time relative to the spike, the y-axis shows the frequencies. The intensities are displayed as a color-coded plot. **(F)** A time-frequency representation is shown where the power for each time is normalized to the mean power of the baseline epoch for that frequency, baseline period: [−1000 ms; −600 ms]. G_TFR_ of the simulated IES demonstrated significant changes between 4 and 30 Hz in the right central sulcus simultaneously with the IES and an increase of power in low frequency band due to increase of the slow-wave amplitude of simulated spikes (0, 5, 10, 15, 20 nAm).

Figure S2 **Transient change in Global Time-Frequency Representation (GTFR) in different scaling factors (800, 400, and 200%)**.

## Abbreviations

BECTS: Benign Epilepsy with Centro-Temporal Spikes
ECoG: ElectroCorticoGraphy
EEG: ElectroEncephaloGraphy
fMRI: functional Magnetic Resonance Imagery
FOS: Fast Optical Signal
FPR: False positive rate
G_TFR_: Global Time-Frequency Representation
HD EEG: High Density EEG
HFOs: High-Frequency Oscillations
I_TFR_: Induced Time-Frequency Representation
IES: Interictal Epileptic Spikes
MUA: MultiUnit Activity
REM: Rapid Eye Movement
TCI: Tansient Cognitive Impairment
TFR: Time-Frequency Representation
sLORETA: Standardized low resolution brain electromagnetic tomography
SSLOFO: Standardized Shrinking LORETA-FOCUSS
WISC: Wechsler Intelligence Scale for Children.
