# Supplementary figures and images for: Local and Distant Dysregulation of Synchronization Around Interictal Spikes in BECTS
Source: Front Neurosci. 2017 Feb 10;11:59. doi: 10.3389/fnins.2017.00059 (PMC5301021; doi:10.3389/fnins.2017.00059)

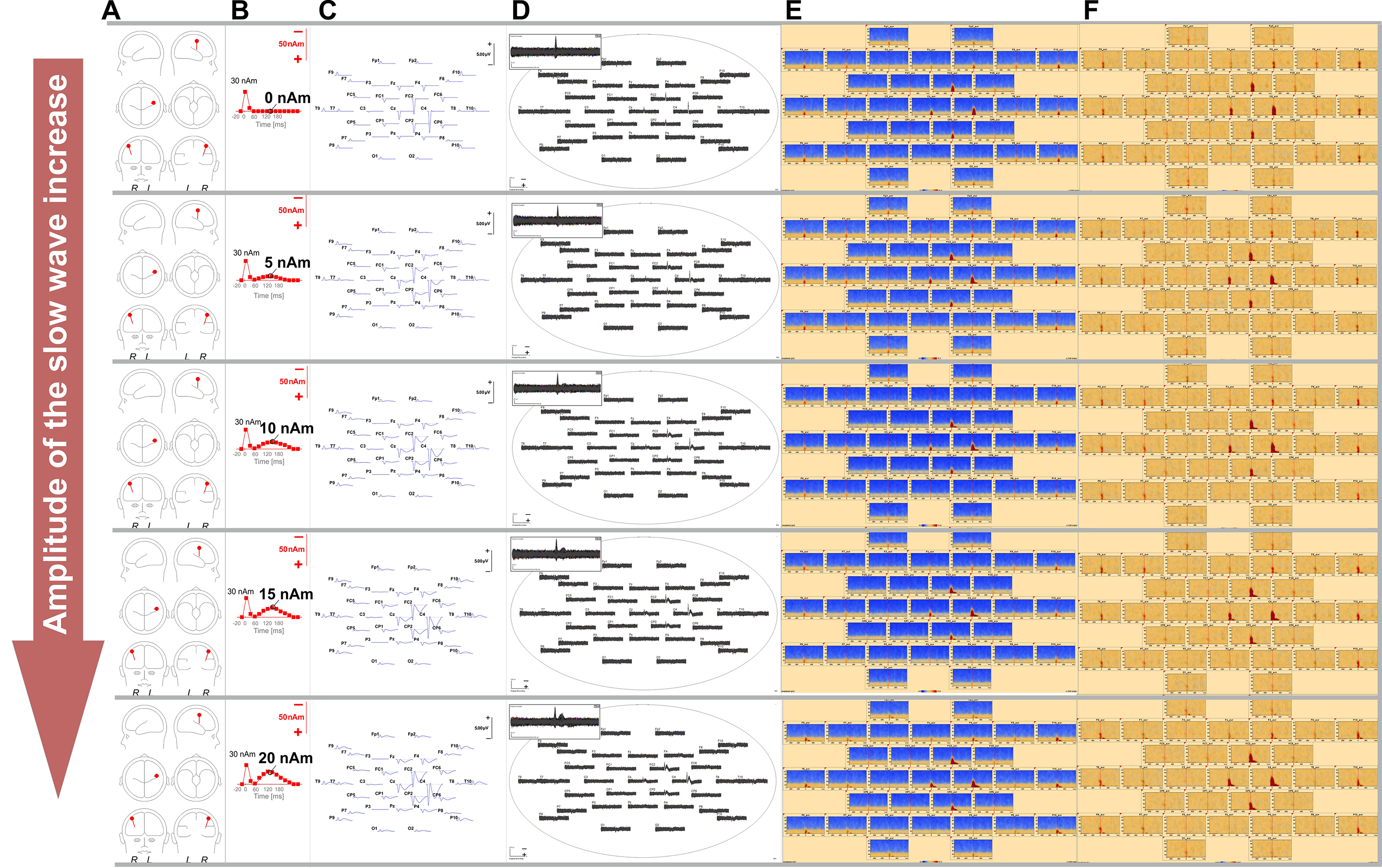

Supplement: Figure S1 — Impact of varying slow wave amplitude on time-frequency analysis. (A) Simulated IES dipole in the right central sulcus, Locations, orientations. (B) Simulated IES with increasing slow wave amplitudes (0, 5, 10, 15, 20 nAm). (C) The simulated scalp IES in average reference format. The solid lines indicate the signal + noise wave forms generated by the dipole sources indicated in (A). (D) Butterfly plot of the simulated spikes, Scalp potentials (33-channel montage) were simulated from dipole in order to correspond to field produced by the generator within right central sulcus. (Up-left) superimposing all channels. (E) The absolute power of time-frequency analysis is displayed. The x-axis shows the time relative to the spike, the y-axis shows the frequencies. The intensities are displayed as a color-coded plot. (F) A time-frequency representation is shown where the power for each time is normalized to the mean power of the baseline epoch for that frequency, baseline period: [−1000 ms; −600 ms]. GTFR of the simulated IES demonstrated significant changes between 4 and 30 Hz in the right central sulcus simultaneously with the IES and an increase of power in low frequency band due to increase of the slow-wave amplitude of simulated spikes (0, 5, 10, 15, 20 nAm). [file Image1.PNG]

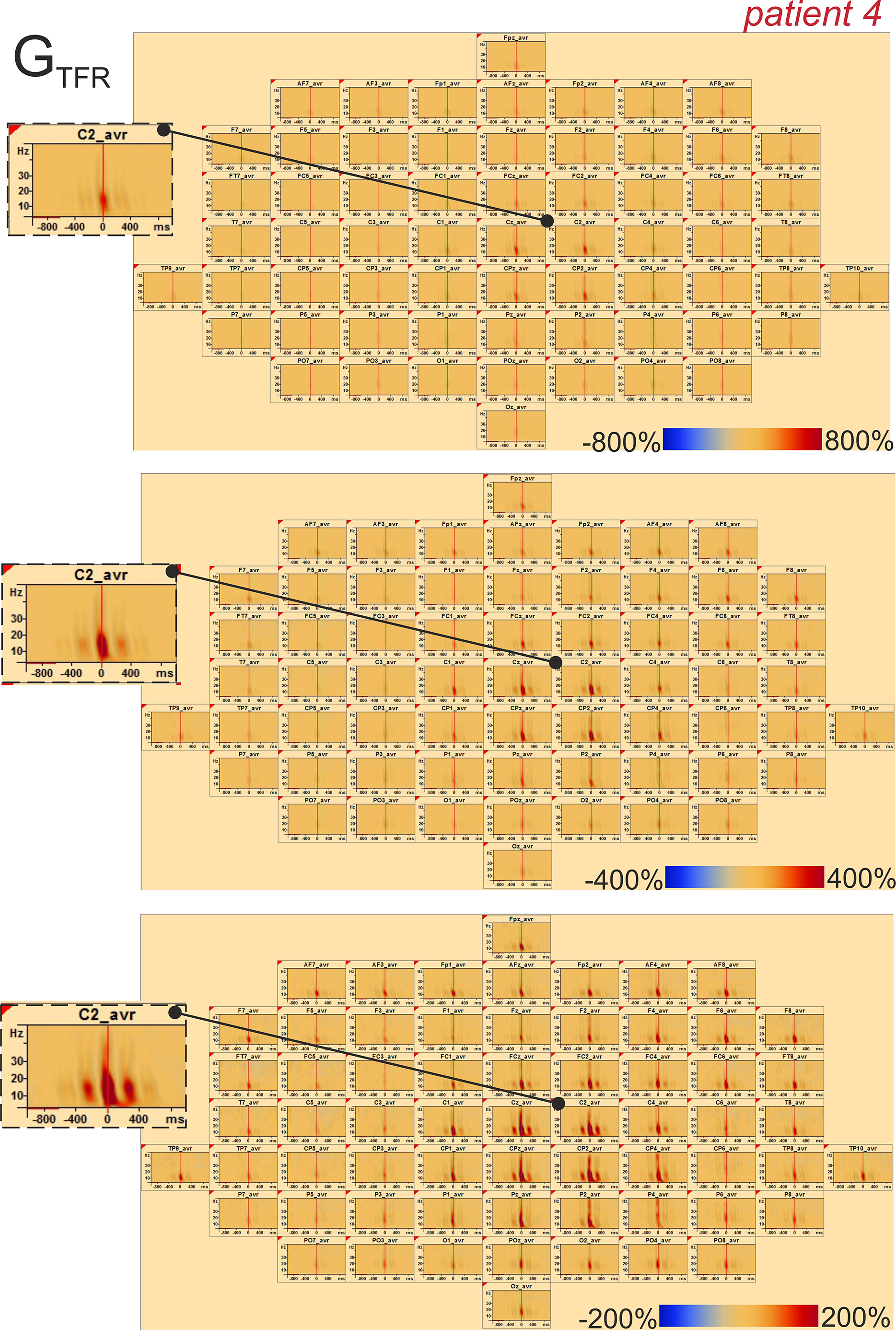

Supplement: Figure S2 — Transient change in Global Time-Frequency Representation (GTFR) in different scaling factors (800, 400, and 200%). [file Image2.PNG]
